# Maternal Separation Differentially Programs Structural and Functional Remodeling of Visceral Adipose Tissue Depots in Mice Exposed to a Post-Weaning High-Fat Diet

**DOI:** 10.3390/ijms27115056

**Published:** 2026-06-03

**Authors:** Javiera Navarrete, Bélgica Vásquez

**Affiliations:** 1 Doctoral Program in Morphological Sciences, Faculty of Medicine, Universidad de La Frontera, Temuco 4780000, Chile; j.navarrete10@ufromail.cl; 2 Center of Excellence in Morphological and Surgical Studies, Universidad de La Frontera, Temuco 4811230, Chile; 3 Department of Basic Sciences, Faculty of Medicine, Universidad de La Frontera, Temuco 4811230, Chile

**Keywords:** maternal separation, high-fat diet, visceral adipose tissue, adipose tissue remodeling, murine model

## Abstract

Visceral adipose tissue (VAT) is a metabolically active organ that undergoes structural and functional remodeling under obesogenic conditions. Early-life stress, such as maternal separation (MS), may modulate these processes, but its depot-specific effects remain poorly characterized. This study aimed to determine whether MS modulates VAT remodeling in response to post-weaning high-fat diet (HFD) exposure in male C57BL/6 mice. Animals underwent MS during the early postnatal period (PND2–16) or remained unmanipulated (UM), and were subsequently fed either a control diet (CD) or an HFD for 16 weeks (groups: UM-CD, UM-HFD, MS-CD, MS-HFD). Visceral adipose tissue was collected and analyzed at PND133. Perigonadal (PGAT), retroperitoneal (RPAT), and mesenteric (MSAT) visceral adipose tissue deposits were analyzed by histology, Picrosirius Red staining, and immunohistochemistry for leptin and UCP-1; apoptosis was assessed by TUNEL assay. HFD induced adipocyte hypertrophy and early inflammatory changes, while MS predominantly affected stromal organization. Collagen remodeling was depot-specific: PGAT showed an adaptive pattern, RPAT exhibited a significant MS×HFD interaction, and MSAT was primarily affected by MS regardless of diet. Leptin immunoreactivity increased with HFD in UM animals but was attenuated in MS mice, particularly in MSAT. UCP-1 signal was low and heterogeneous, without clear morphological browning. Apoptosis increased in MSAT under MS-HFD conditions. These findings indicate that early-life stress programs depot-specific VAT remodeling, with MSAT emerging as particularly susceptible to obesogenic challenge.

## 1. Introduction

The Visceral adipose tissue (VAT) is recognized as a metabolically active organ that integrates energy storage, endocrine secretion, and immune regulation [1,2]. In contrast to subcutaneous adipose tissue, visceral depots exhibit greater metabolic and inflammatory sensitivity to energy surplus, making them key contributors to the development of obesity-related complications [3,4]. Nevertheless, visceral fat depots are not a homogeneous entity: perigonadal (PGAT), retroperitoneal (RPAT), and mesenteric (MSAT) adipose tissues differ in embryological origin, anatomical features, vascular drainage, and exposure to local metabolic cues. Consequently, their response to an obesogenic environment is depot-specific and may be further modulated by early-life metabolic programming factors [5,6].

Expansion of visceral fat induced by a high-fat diet (HFD) is accompanied by extensive remodeling, including adipocyte hypertrophy, immune cell infiltration, and reorganization of the extracellular matrix (ECM) [7,8]. Histologically, this process is characterized by interstitial inflammatory infiltrates and the presence of crown-like structures (CLS), which are defined as aggregates of macrophages arranged in a semicircular or ring-like pattern around degenerating or dying adipocytes. These features are associated with the phagocytosis of lipid debris, and CLS are well-established morphological hallmarks of adipose tissue inflammation and metabolic dysfunction [9,10]. In parallel, progressive deposition of collagen fibers leads to fibrosis, restricting healthy tissue expansion and diminishing structural plasticity [7,11]. Cell death-related processes constitute an additional component of adipose tissue remodeling, contributing to immune cell recruitment and perpetuation of inflammatory signaling [12].

Maternal separation (MS) is a widely employed model to investigate the impact of early-life adversity on metabolic programming. This form of postnatal stress induces persistent alterations in the hypothalamic–pituitary–adrenal (HPA) axis and glucocorticoid sensitivity, thereby modulating lipid metabolism, adipogenesis, and adipose tissue inflammation later in life [13,14]. In this context, MS may serve as a programming factor that conditions the magnitude and nature of adipose tissue responses to subsequent metabolic challenges, such as chronic exposure to an HFD [15,16].

Adipose tissue expansion is also accompanied by changes in its secretory profile and functional capacity, which may be shaped by a history of early-life stress. Leptin, beyond its established role in energy homeostasis, exerts local immunomodulatory effects; alterations in these effects are associated with inflammation and adipose tissue dysfunction in obesogenic settings [17,18]. Uncoupling protein-1 (UCP-1), on the other hand, reflects the metabolic plasticity of white adipose tissue under energy challenges, and alterations in its expression within visceral fat depots may signal compromised functional adaptability [19,20]. Nevertheless, the degree to which early-life stress programming differentially modulates these functional and structural responses across visceral adipose depots remains unclear.

The aim of this study was to determine whether early-life stress induced by MS conditions the structural and functional remodeling of visceral adipose tissue, specifically perigonadal (PGAT), retroperitoneal (RPAT), and mesenteric (MSAT) adipose tissues, following post-weaning HFD exposure in C57BL/6 mice.

## 2. Results

### 2.1. Histological Findings

**Figure 1:**
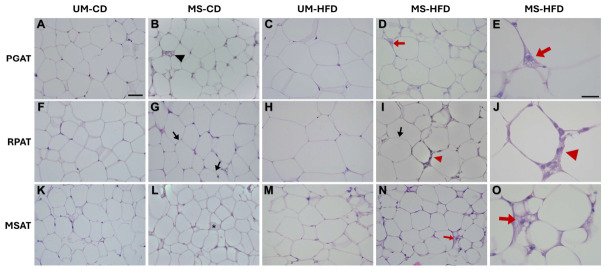
Histological alterations in visceral adipose depots induced by maternal separation and high-fat diet. PGAT (**A**–**E**), RPAT (**F**–**J**), and MSAT (**K**–**O**) from UM-CD, MS-CD, UM-HFD, and MS-HFD groups. HFD groups (**C**,**H**,**M**) show histological features consistent with adipocyte enlargement, in agreement with previously reported morphometric findings from the same experimental cohort. MS-CD groups (**B**,**G**) display stromal alterations characterized by increased interstitial mononuclear cell infiltration and greater stromal visibility. In MS-HFD groups (**D**,**I**,**N**), adipocyte deformation and inflammatory infiltration are more pronounced. Panels (**E**,**J**,**O**) show higher-magnification views of MS-HFD tissue, mononuclear inflammatory cell infiltration and structures morphologically compatible with crown-like structures (CLS) in each depot. Black arrows: irregular adipocyte contours; asterisk: intracytoplasmic eosinophilic inclusions; black arrowhead: blood vessel; red arrows: macrophage-like cells; red arrowhead: structure morphologically compatible with an incipient CLS. Scale bars: 40 µm (**A**–**D**,**F**–**I**,**K**–**N**); 20 µm (**E**,**J**,**O**). H&E staining.

Histological examination of the three visceral adipose depots using hematoxylin and eosin (H&E) staining revealed morphological differences associated with HFD and MS. Quantitative analyses previously performed in the same experimental cohort included adipose depot mass, adipocyte sectional area (SA), and general tissue architecture associated with adipocyte size distribution patterns [21]. Therefore, the present study focused on complementary histopathological features not previously characterized, including inflammatory infiltrates, adipocyte deformation, vascular alterations, and crown-like structures (CLS), providing additional information regarding adipose tissue remodeling (Figure 1).

Across all depots, the UM-CD group (Figure 1A,F,K) exhibited preserved histological architecture characteristic of visceral white adipose tissue. Adipocytes displayed relatively uniform size, smooth contours, and a single unilocular lipid vacuole, with flattened nuclei positioned at the cell periphery. The interadipocyte stroma was sparse, and blood vessels of normal caliber were occasionally observed. The homogeneous adipocyte appearance was consistent with preserved visceral white adipose tissue morphology.

Exposure to HFD was predominantly associated with adipocyte hypertrophy. In UM-HFD animals, adipocytes displayed histological features consistent with enlargement and altered tissue organization, accompanied by thinning of the cytoplasmic rim. These alterations were most pronounced in the RPAT (Figure 1H), where adipocytes exhibited marked polygonalization resulting from increased tissue compaction. In some microscopic fields of RPAT (Figure 1I,J) and MSAT (Figure 1N,O), scattered mononuclear cells and structures morphologically compatible with early CLS around collapsed adipocytes were observed.

In the MS-CD group, morphological changes were predominantly observed in the stromal compartment. In PGAT, greater visibility of interstitial blood vessels (Figure 1B) and more frequent scattered mononuclear cells with macrophage-like morphology were noted. Additionally, mild heterogeneity in adipocyte size and a more prominent and slightly thickened cytoplasmic rim were observed compared to the UM-CD group. In this group, adipocytes with irregular contours were also identified, characterized by wavy or flattened segments at intercellular contact surfaces (Figure 1G).

When MS was combined with HFD (MS-HFD), morphological changes were more evident across all three depots (Figure 1D,E,I,J,N,O). Adipocytes exhibited marked hypertrophy, greater heterogeneity in cell size, and a higher proportion of deformed adipocytes with distorted contours, undulating plasma membranes, and partially flattened surfaces. This deformation pattern was associated with increased tissue compaction and adipocyte expansion. Occasional discrete intracytoplasmic eosinophilic inclusions, compatible with myelin-like structures, were also observed in PGAT (Figure 1L).

Clear differences were observed among the visceral adipose depots. PGAT primarily displayed stromal alterations associated with MS, whereas RPAT demonstrated more pronounced adipocyte hypertrophy in response to HFD and the presence of CLS in some microscopic fields. MSAT exhibited the most prominent inflammatory profile, characterized by a marked interstitial mononuclear infiltrate and a higher frequency of structures morphologically compatible with CLS in the MS-HFD groups.

In summary, the visceral adipose depots exhibited differential morphological patterns: predominant adipocyte hypertrophy with HFD, and more evident stromal alterations in animals exposed to MS.

### 2.2. Picrosirius Red

**Figure 2:**
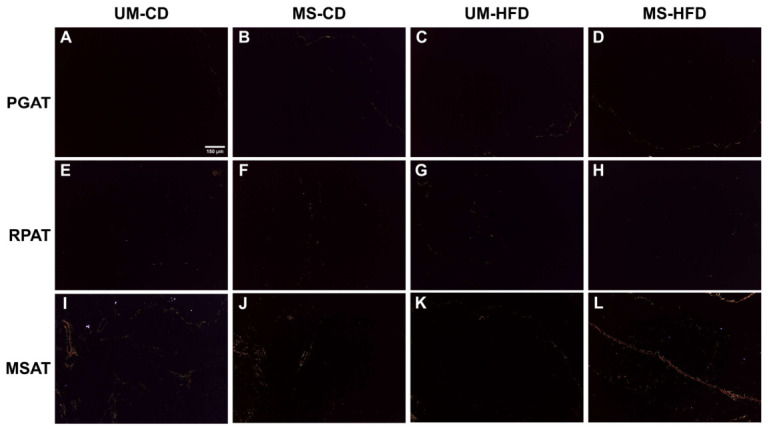
Representative polarized light micrographs of Picrosirius Red (PSR)-stained visceral adipose tissue from the perigonadal (PGAT), retroperitoneal (RPAT), and mesenteric (MSAT) adipose depots of male C57BL/6 mice subjected to maternal separation and post-weaning high-fat diet exposure. Panels (**A**–**D**): PGAT; panels (**E**–**H**): RPAT; panels (**I**–**L**): MSAT. Groups correspond to unmanipulated, control diet (UM-CD); maternal separation, control diet (MS-CD); unmanipulated, high-fat diet (UM-HFD); and maternal separation, high-fat diet (MS-HFD). Red-orange birefringent signals indicate type I collagen fibers, while yellow-green signals indicate type III collagen fibers under polarized light. Scale bar: 150 μm for all panels.

**Figure 3:**
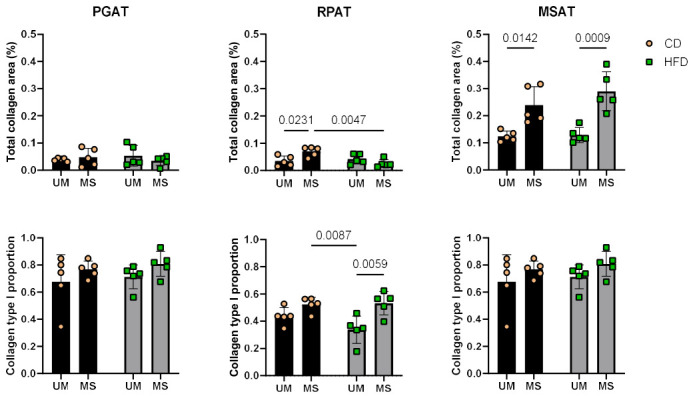
Quantitative analysis of collagen in visceral adipose depots stained with Picrosirius Red and visualized under polarized light. (**Upper panels**) show total collagen area (%) and (**lower panels**) show the normalized proportion of type I collagen fibers in perigonadal (PGAT), retroperitoneal (RPAT), and mesenteric (MSAT) depots. CD: control diet; HFD: high-fat diet; MS: maternal separation; UM: unmanipulated. Data are presented as mean ± SD with individual values for each animal. Statistical analysis was performed using two-way ANOVA. *p* < 0.05 is considered statistically significant.

**Figure 4:**
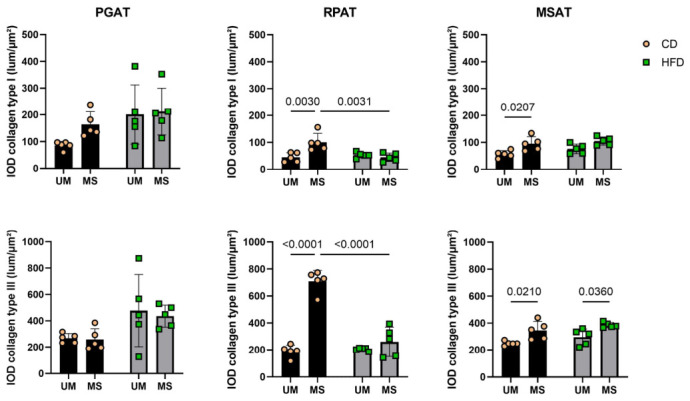
Quantification of birefringent collagen fibers in visceral adipose depots stained with Picrosirius Red and visualized under polarized light. (**Upper panels**) show the integrated optical density (IOD) of type I collagen fibers and (**lower panels**) show the IOD of type III collagen fibers in perigonadal (PGAT), retroperitoneal (RPAT), and mesenteric (MSAT) depots. CD: control diet; HFD: high-fat diet; MS: maternal separation; UM: unmanipulated. Data are presented as mean ± SD with individual values for each animal. Statistical analysis was performed using two-way ANOVA. *p* < 0.05 is considered statistically significant.

Given the histologically observed stromal alterations, collagen organization within the stromal vascular compartment was assessed using Picrosirius Red staining under polarized light. Representative polarized micrographs showed birefringent collagen fibers predominantly distributed around adipocytes and within connective tissue septa across all visceral adipose depots (Figure 2). PGAT (Figure 2A–D) generally exhibited weaker birefringent signals, whereas RPAT (Figure 2E–H) showed intermediate and heterogeneous signal intensity. In contrast, MSAT (Figure 2I–L) displayed a more intense birefringent signal pattern across the analyzed fields. These qualitative observations suggested depot-dependent differences in collagen organization and prompted further quantitative evaluation of collagen content and fiber characteristics (Figure 3 and Figure 4).

When comparing depots, MSAT consistently showed a higher percentage of total collagen area than PGAT and RPAT, indicating a greater ECM content even under basal conditions (Figure 3).

With respect to collagen fiber packing and maturity, the integrated optical density (IOD) of both fiber populations, reflecting collagen birefringence intensity and fiber packing characteristics, increased in animals fed HFD (Figure 4). This was confirmed by a significant main effect of diet for both type I fibers (*p* = 0.0259; 24.3% of total variance) and type III fibers (*p* = 0.0109; 33.84% of total variance) (Table 1).

In RPAT, collagen remodeling was more pronounced and dependent on the combination of experimental factors. Animals exposed to MS showed increased total collagen content under CD, an effect that was further amplified by HFD, yielding a significant interaction between diet and MS (*p* = 0.0041). This was accompanied by increased intensity of both type I and type III fibers, with a significant interaction between the two factors (*p* = 0.0032 for type I fibers; *p* < 0.0001 for type III fibers) (Table 1). Notably, RPAT exhibited relatively high optical density in type III collagen fibers, indicating a greater contribution associated with early matrix remodeling (Figure 4). Together, these findings indicate that collagen remodeling in RPAT depends on the convergence of early metabolic programming and nutritional challenge, rather than on the isolated effect of either factor.

In MSAT, MS was the primary determinant of collagen remodeling. Animals subjected to MS exhibited higher total collagen content under both dietary conditions (*p* < 0.0001; Table 1 In parallel, HFD was associated with a compositional shift toward a higher relative proportion of type III fibers (*p* = 0.0273; Table 1). These findings indicate that MS exerts a predominant effect on ECM organization in MSAT, suggesting that early metabolic programming may predispose this depot to structural remodeling even in the absence of dietary challenge.

In summary, visceral adipose depots exhibited distinct patterns of collagen organization: relative stability in PGAT, changes dependent on the interaction between MS and HFD in RPAT, and a predominant effect of MS in MSAT.

### 2.3. Immunohistochemistry

#### 2.3.1. Leptin

**Figure 5:**
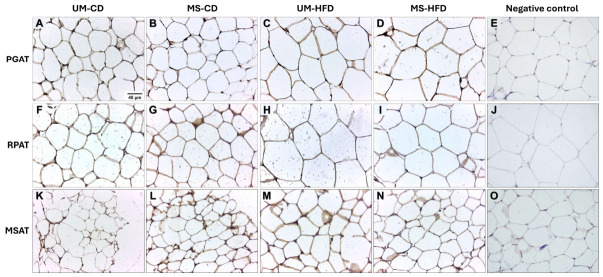
Representative leptin immunostaining in the perigonadal (PGAT), retroperitoneal (RPAT), and mesenteric (MSAT) visceral adipose depots of male C57BL/6 mice subjected to maternal separation and post-weaning high-fat diet exposure. Panels (**A**–**E**): PGAT; panels (**F**–**J**): RPAT; panels (**K**–**O**): MSAT. Within each adipose depot, panels correspond to unmanipulated, control diet (UM-CD); maternal separation, control diet (MS-CD); unmanipulated, high-fat diet (UM-HFD); maternal separation, high-fat diet (MS-HFD); and negative control, respectively. Brown DAB precipitate indicates leptin immunoreactivity. Scale bar: 40 μm for all panels.

**Figure 6:**
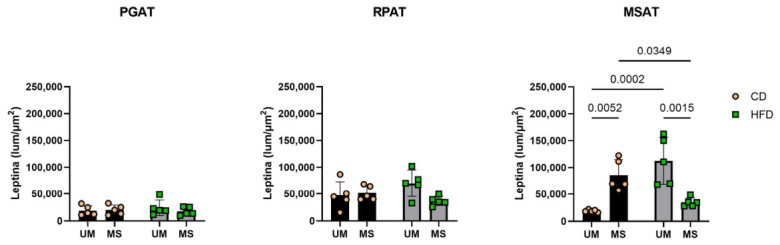
Integrated optical density (IOD) of leptin immunostaining in perigonadal (PGAT), retroperitoneal (RPAT), and mesenteric (MSAT) adipose tissue deposits from male C57BL/6 mice subjected to maternal separation and post-weaning high-fat diet exposure. CD: control diet; HFD: high-fat diet; MS: maternal separation; UM: unmanipulated. Data are presented as mean ± SD with individual values for each animal. Statistical analysis was performed using two-way ANOVA. *p* < 0.05 is considered statistically significant.

Adipose tissue expansion entails not only alterations in ECM organization but also functional adaptations in adipocyte secretory activity. Representative leptin immunostaining revealed a positive signal appearing as a brown DAB precipitate with cytoplasmic localization, confined to the thin cytoplasmic rim surrounding the adipocyte lipid vacuole (Figure 5). Negative controls showed no detectable nonspecific staining, confirming the specificity of the immunohistochemical reaction. Visual differences in staining intensity were observed among visceral adipose depots and experimental groups. PGAT (Figure 5A–D) and RPAT (Figure 5E–H) generally exhibited weaker immunoreactive signals, whereas more evident staining patterns were observed in MSAT sections (Figure 5I–L). These qualitative observations prompted subsequent quantitative evaluation of leptin immunoreactivity (Figure 6).

In PGAT, immunostaining revealed no significant differences among groups, nor any effects attributable to diet, MS, or their interaction. Similarly, RPAT showed no significant group differences in immunostaining; however, a significant interaction between diet and MS was observed. In MSAT, UM animals showed a significant increase in leptin immunolabeling in response to HFD, whereas MS animals exhibited reduced leptin immunoreactivity under the same nutritional challenge (Figure 6). This effect was most pronounced in MSAT, where the diet × MS interaction accounted for 65.67% of the total variance (Table 2).

#### 2.3.2. UCP-1

**Figure 7:**
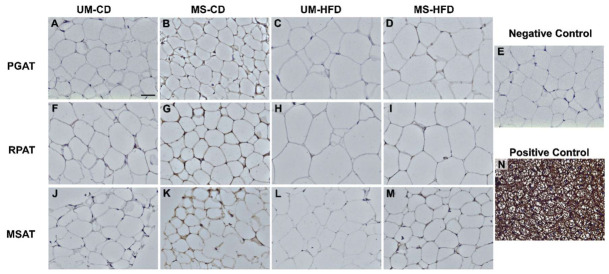
UCP-1 Immunostaining in visceral adipose tissue of the perigonadal (PGAT), retroperitoneal (RPAT), and mesenteric (MSAT) adipose tissue deposits from male C57BL/6 mice subjected to maternal separation and post-weaning high-fat diet exposure. Panels (**A**–**D**): PGAT; panels (**F**–**I**): RPAT; panels (**J**–**M**): MSAT; (**E**) negative control; (**N**) positive control. UM-CD: unmanipulated, control diet; MS-CD: maternal separation, control diet; UM-HFD: unmanipulated, high-fat diet; MS-HFD: maternal separation, high-fat diet. Brown staining indicates UCP-1 immunoreactivity in brown adipose tissue. Scale bar: 40 μm for all panels.

In the PGAT, RPAT, and MSAT adipose tissue depots, immunostaining for UCP-1 did not reveal the presence of multilocular adipocytes or morphological patterns consistent with browning in any of the experimental groups (Figure 7). In contrast, the positive control, consisting of brown adipose tissue, showed the characteristic immunoreactivity for this marker, visualized as a brown 3,3′-diaminobenzidine (DAB) precipitate with cytoplasmic distribution, outlining the multiple lipid vacuoles characteristic of multilocular brown adipocytes (Figure 7N). Consequently, the tissue architecture observed in all groups consistently corresponded to unilocular white adipose tissue.

However, in all three adipose depots of animals from the MS-CD group, and to a lesser extent in the MS-HFD group, faint, heterogeneous, and irregular cytoplasmic immunostaining for UCP-1 was detected, localized to the peripheral cytoplasmic rim of adipocytes; this pattern was absent in UM animals (Figure 7B,D,G,I,K,M). The observed immunoreactivity was restricted to unilocular adipocytes and was not accompanied by morphological characteristics typically associated with beige or brown adipocyte differentiation, such as multilocular lipid droplets or reduced adipocyte size.

Due to the low signal intensity and the absence of morphological changes compatible with beige or brown adipocytes, this immunoreactivity was not subjected to quantification.

### 2.4. TUNEL Assay

DNA fragmentation was evaluated using the TUNEL assay in representative samples of PGAT and MSAT. The analysis was conducted on a limited number of samples and was therefore interpreted descriptively.

In PGAT, the TUNEL signal was virtually absent in the analyzed fields. The UM-CD, UM-HFD, and MS-CD groups exhibited no detectable TUNEL-positive nuclei, whereas in MS-HFD, isolated TUNEL-positive nuclei were identified, corresponding to approximately 1.35 nuclei per 100 adipocytes.

In contrast, occasional TUNEL-positive nuclei were observed in MSAT, even under basal conditions. In UM-CD, the frequency was approximately 0.97 positive nuclei per 100 adipocytes, a value that remained comparable in UM-HFD (1 per 100 adipocytes). The MS-HFD group showed the highest frequency of TUNEL-positive nuclei, reaching approximately 1.62 per 100 adipocytes. Quantifiable images could not be obtained for the MS-CD group in this depot; therefore, no estimates are available for this condition.

Overall, TUNEL-detectable DNA fragmentation was low in both adipose depots but exhibited a differential pattern between depots. While PGAT showed a virtually absent signal, MSAT demonstrated detectable basal TUNEL positivity that tended to increase under the combination of MS and HFD.

## 3. Discussion

The expansion of visceral adipose tissue induced by excess energy requires coordinated adaptation among adipocytes, the vasculature, and the ECM to accommodate the increase in cell volume without compromising tissue function [22]. In murine models of diet-induced obesity, this process manifests primarily as adipocyte hypertrophy, stromal remodeling, and immune cell infiltration, processes that contribute to the establishment of a metabolically active and pro-inflammatory microenvironment [23,24].

Our findings extend this model by demonstrating that early-life metabolic programming induced by MS modulates this response differentially across visceral depots, conditioning both the structural organization and the functional response of VAT.

*Adipose tissue expansion induced by HFD was accompanied by adipocyte hypertrophy, morphological changes characteristic of tissue expansion, and early signs of stromal inflammatory activation.*

Previous analyses using the same experimental cohort demonstrated increased visceral adipose tissue mass, adipocyte hypertrophy, and adiposity index in response to HFD [21]. In addition, alterations in body weight had been previously reported in studies performed using the same experimental cohort. In this context, histological analysis of visceral adipose depots allowed us to explore how these systemic and tissue-level alterations are associated with changes in the stromal and inflammatory microenvironment of adipose tissue.

Adipocytes exhibited marked hypertrophy, accompanied by heterogeneity in cell size and a higher proportion of deformed cells with distorted contours, undulating plasma membranes, and partially flattened surfaces. This morphological pattern is consistent with tissue compaction phenomena associated with adipose expansion, where uneven growth of adjacent adipocytes and mechanical constraints imposed by the surrounding stroma can alter the cellular architecture of adipose tissue and generate cell deformations characteristic of expanded adipose tissue [25,26]. In hypertrophied adipose tissue, these morphological alterations often coexist with changes in ECM organization and activation of local inflammatory responses [27].

Occasionally, discrete intracytoplasmic eosinophilic inclusions compatible with myelin-like structures, previously described in adipocytes under lipid stress and during tissue remodeling associated with obesity, were also observed in PGAT [28,29,30].

Consistent with these structural alterations, increased mononuclear inflammatory cell infiltration in the interstitial stroma was observed, whereas CLS was infrequent. This pattern aligns with observations in diet-induced obesity models, where inflammatory cell recruitment may precede the appearance of morphologically identifiable CLS-like structures, representing an early stage of the adipose tissue inflammatory response to the tissue stress generated by adipocyte hypertrophy [23,24,31].

Beyond their classical pro-inflammatory role, macrophages contribute to broader adipose tissue remodeling processes during obesity. Recent studies using single-cell and single-nucleus approaches further suggest that macrophage accumulation may occur in parallel with alterations involving neural and vascular components of adipose tissue microenvironments [32,33,34,35]. However, these mechanisms were not directly evaluated in the present study. Collectively, the observed morphological alterations and immune heterogeneity suggest that adipose expansion in this model involves coordinated inflammatory and stromal responses whose magnitude may differ across visceral depots.

*Extracellular matrix remodeling in visceral adipose tissue is not uniform across depots but instead reflects the interplay between diet-induced adipose expansion, early-life metabolic programming, and the intrinsic physiological characteristics of each compartment.*

Structural alterations in adipose tissue were further evidenced by changes in the fibrillar organization of collagen, with depot-specific patterns. In PGAT, the observed modifications suggest adaptive ECM reorganization rather than an increase in total collagen content. Adipocyte hypertrophy may generate mechanical stress and localized microhypoxia, promoting fibroblast activation and macrophage recruitment, which actively modulate collagen organization through the release of cytokines and matrix-remodeling enzymes [33,36]. These changes likely reflect adjustments in fiber packing or maturation, representing an early phase of stromal remodeling during expansion induced by energy excess [37].

In contrast, in RPAT, collagen remodeling showed a response dependent on the interaction between diet and MS, suggesting that early-life metabolic programming shapes the stromal response to nutritional challenge. In relation to this, MS may induce persistent alterations in the HPA axis and glucocorticoid signaling, which in turn modulate the metabolic and inflammatory responses of adipose tissue at later life stages [38,39]. When this previously programmed tissue is exposed to HFD, adipose expansion creates a microenvironment of mechanical stress and hypoxia that, combined with macrophage infiltration and stromal cell activation, promotes more pronounced remodeling of the stromal vascular compartment [36,37,40]. The higher signal of thin green-yellow birefringent fibers observed in this depot also suggests a greater contribution of thin fibers associated with early phases of ECM reorganization.

In MSAT, MS was the primary determinant of collagen remodeling, irrespective of dietary intervention. This observation aligns with the higher basal collagen content detected in this depot relative to PGAT and RPAT, suggesting that the MSAT possesses an intrinsically denser ECM architecture and may therefore be more susceptible to stromal remodeling processes.

These results suggest that MS programs a baseline ECM reorganization in this depot that manifests independently of nutritional challenge, likely through the same HPA axis disruption and glucocorticoid signaling pathways described for RPAT [15,41]. Building on this programmed foundation, HFD further modulated the fibrillar composition, increasing the relative proportion of type III collagen, a pattern consistent with early stromal remodeling during tissue expansion [40,42]. This combination of effects is particularly relevant in MSAT due to its direct drainage into the portal circulation, which exposes this depot early to lipids, cytokines, and intestinal mediators, rendering it especially sensitive to stromal remodeling associated with visceral obesity [43]. Consequently, the structural alterations observed in MSAT acquire systemic significance, as the portal circulation represents the direct communication pathway between this depot and the liver. ECM remodeling in MSAT, together with increased apoptosis under MS-HFD conditions, may promote greater release of free fatty acids and proinflammatory cytokines into the portal vein, generating a lipotoxic and chronic inflammatory environment in the hepatic parenchyma [44]. This mechanism is consistent with the findings of del Sol et al. [45], who demonstrated in the same experimental model that the combination of MS and a high-calorie diet induced steatosis, mixed inflammatory infiltrate, and hepatocellular ballooning, with a significantly higher NAS in the MS-HFD group, suggesting progression toward NASH at early stages of postnatal development.

Beyond the liver, dysfunctional MSAT may also compromise pancreatic function. Chronic exposure of the pancreas to free fatty acids and inflammatory mediators via the shared portal drainage promotes beta-cell lipotoxicity, impairing insulin secretion and contributing to insulin resistance, which are central mechanisms in the pathogenesis of type 2 diabetes [46,47]. In similar experimental models, the combination of early-life stress and a high-calorie diet has been shown to induce fasting hyperinsulinemia and elevate the HOMA-IR index, further supporting the notion that MSAT programmed by early-life stress actively contributes to impaired glucose homeostasis [48].

Taken together, these findings suggest that structural and functional alterations in MSAT may constitute an early link in the cascade of events that, via the portal axis, contribute to hepatic dysfunction, impaired glucose homeostasis, and insulin resistance, processes that collectively converge in the development of metabolic syndrome.

*Diet-induced adipose tissue expansion is associated with alterations in leptin expression dependent on early-life history, characterized by an enhanced response in UM animals and an opposite response in MS animals, reflecting differential secretory regulation of VAT.*

Adipose tissue expansion involves not only changes in ECM organization but also functional adjustments in adipocyte secretory activity. In UM animals, HFD was associated with increased leptin immunoreactivity in PGAT and RPAT adipocytes, a finding consistent with the greater adiposity and adipocyte hypertrophy previously described in this experimental model [21]. Leptin expression is closely correlated with adipocyte size and adipose tissue expansion, reflecting the capacity of adipocytes to respond to energy excess and participate in the regulation of metabolic homeostasis [49]. Furthermore, this adipokine plays a key role in adipocyte-immune cell communication, contributing to the establishment of a low-grade inflammatory state in expanded adipose tissue [50].

In contrast, in animals subjected to MS, the leptin response to HFD showed a distinct pattern, particularly in the RPAT and MSAT. This pattern suggests that early-life metabolic programming may influence adipokine regulation in VAT. Several studies have demonstrated that early-life stress can induce persistent alterations in leptin signaling and adipose metabolism, altering the response to obesogenic diets at later life stages [38,39,51].

The decrease in leptin immunoreactivity observed in MS animals under HFD, particularly in MSAT, could result from the combination of several factors operating simultaneously. Because mature adipocytes contain a large unilocular lipid droplet occupying most of the cellular volume, leptin immunoreactivity should be interpreted as a local distribution pattern of the detected signal rather than as a direct measure of intracellular leptin synthesis or secretion. First, persistent HPA axis reprogramming induced by early postnatal stress could influence leptin immunoreactivity patterns within visceral adipose tissue independently of tissue energy status [16,51], an effect consistent with evidence supporting leptin as a biomarker sensitive to chronic stress [52]. Additionally, the increased collagen density observed in MSAT under MS conditions could limit adipocyte secretory capacity by altering intercellular communication and diffusion of paracrine signals, contributing to a dissociation between adipose tissue expansion and leptin immunoreactivity patterns [53]. Extracellular matrix remodeling may also influence the local distribution of the immunoreactive signal and should therefore be considered when interpreting these findings.

The biological consequences of this reduction in leptin levels would act concurrently. Leptin exerts a tonic inhibitory effect on AgRP neurons in the hypothalamic arcuate nucleus, which are orexigenic neurons whose activation stimulates food intake and decreases energy expenditure [54,55]. Therefore, reduced leptin immunoreactivity in MSAT could relieve this inhibition, impair hypothalamic signaling, and sustain a positive energy balance. Moreover, hypoleptinemia may stimulate glucocorticoid production, which in turn could further suppress leptin synthesis and potentially contribute to a self-reinforcing feedback mechanism associated with long-term neuroendocrine alterations induced by early postnatal stress [38,56]. In this context, the concomitant presence of an increased inflammatory infiltrate and structures morphologically compatible with CLS in MSAT suggests that inflammation progresses through leptin-independent mechanisms, potentially associated with inflammatory cell recruitment and cytokine-mediated mechanisms and the subsequent release of cytokines such as TNF-α and IL-6 [57].

Taken together, these findings indicate that animals exposed to early postnatal stress and a high-calorie diet may exhibit increased food intake, greater adiposity, and impaired body weight regulation, thereby heightening susceptibility to obesity and associated metabolic complications.

*Exploratory TUNEL evaluation revealed low-frequency TUNEL-positive nuclei across adipose depots, with a more evident signal in MSAT and a tendency toward increased labeling under combined MS and HFD conditions.*

In this context, the inflammatory and metabolic alterations accompanying adipose tissue expansion may also be linked to cellular stress and DNA fragmentation-related processes involved in adipose tissue remodeling during obesity. DNA fragmentation was assessed by TUNEL assay in a limited number of samples for exploratory purposes; therefore, these findings should be interpreted with caution and regarded as preliminary and hypothesis-generating rather than definitive.

In PGAT, TUNEL positivity was virtually absent in most groups, except for isolated TUNEL-positive nuclei in the MS-HFD group, which may reflect a predominantly adaptive expansion phase in this depot. In contrast, in MSAT, TUNEL-positive nuclei were detectable even under control conditions, with a tendency to increase under the combined influence of MS and HFD. This pattern is consistent with other findings previously described in this depot, including greater collagen remodeling, an inverted leptin response under HFD, and increased macrophage infiltration in the stromal vascular fraction. Together, these features constitute a microenvironment of progressive metabolic and inflammatory vulnerability to the combined effects of early-life programming and nutritional challenge, mediated by mechanisms related to portal exposure and glucocorticoid programming described above for this depot.

In models of diet-induced obesity, TUNEL positivity has been described as a focal and low-frequency event during the early stages of tissue expansion [31,58]. Thus, the values observed in MSAT may represent an early phase of adipocyte dysfunction with potential implications for the fibro-inflammatory remodeling of this depot, a hypothesis that warrants further investigation in studies with larger sample sizes.

*The absence of morphological evidence of browning across all groups contrasts with the weak UCP-1 immunoreactivity observed in maternally separated animals, suggesting altered adipose tissue metabolic plasticity rather than active browning.*

None of the visceral fat depots evaluated showed multilocular adipocytes or morphological features indicative of browning, a finding consistent with the limited capacity of visceral depots to activate thermogenic programs in response to adrenergic stimuli compared with subcutaneous depots [59,60]. However, in MS-CD animals, and to a lesser extent in the MS-HFD group, weak, heterogeneous, and irregular UCP-1 immunoreactivity was observed across all three visceral depots. This immunoreactivity was attenuated, but not eliminated, under HFD, remaining above levels observed in UM animals.

The absence of morphological changes consistent with functional beige adipocytes suggests that this signal does not reflect an active browning process but may instead represent subtle alterations associated with early-life programming. In this context, the weak and heterogeneous distribution of the signal further supports the interpretation that UCP-1 immunoreactivity occurred in the absence of overt morphological remodeling toward a beige adipocyte phenotype. Given that UCP-1 expression is influenced by sympathetic signaling through the β-AR/cAMP/PKA pathway [61]. Razzoli et al. [62] demonstrated that stress can activate UCP-1 expression in adipose tissue through sympathetic mechanisms. Previous studies have also shown that early-life stress induces long-term alterations in adipose tissue function and metabolic regulation [51,63]. Therefore, the weak UCP-1 immunoreactivity observed in maternally separated animals may reflect subtle alterations in adipose tissue metabolic responsiveness associated with early-life programming rather than evidence of active browning.

Taken together, these results demonstrate that visceral adipose tissue does not respond uniformly to the combination of early-life stress and obesogenic diet. Instead, each depot integrates these factors in a manner determined by its physiological identity and programming history. This depot-specific heterogeneity has important implications for understanding how early adversity increases metabolic vulnerability in adulthood and underscores the need to consider each adipose depot as an independent functional unit when designing preventive and therapeutic strategies for populations exposed to early-life stress.

This study has several limitations that should be considered when interpreting the results. First, the analysis was restricted to male mice, which limits the extrapolation of findings to females, whose responses to early-life stress and obesogenic diet may differ. Additionally, although the morphological and immunohistochemical approach enabled precise characterization of structural and secretory changes in visceral adipose tissue, it did not allow identification of the underlying molecular mechanisms. In addition, inflammatory infiltrates and CLS were identified based on histological morphology alone; therefore, the absence of specific macrophage markers did not permit definitive immunophenotypic characterization. Finally, the evaluation at a single time point does not permit the determination of whether the observed alterations represent the onset of a progressive trajectory or a transient phenomenon.

These findings open several avenues for further research. Including female animals would help determine whether sex-related differences exist in depot-specific vulnerability. Complementary molecular analyses, including quantification of plasma leptin, cytokine profiling, assessment of gene expression for inflammatory and fibrosis markers, and characterization of the systemic metabolic profile, would facilitate linking morphological observations to measurable functional alterations. Finally, longitudinal assessment at multiple time points would help determine whether these alterations progress toward established metabolic dysfunction later in life.

## 4. Materials and Methods

### 4.1. Animals

Adipose tissue samples were obtained from male C57BL/6 mice enrolled in an experimental model developed within the UFRO-DIUFRO project (code DI24-0029), originally designed to evaluate the combined effects of early-life stress induced by maternal separation and post-weaning HFD exposure on pancreatic structure and function. Additional analyses of adipose tissue were performed using tissues collected from the same experimental cohort, allowing expansion of the scientific scope without increasing the total number of animals used, in accordance with the principles of reduction and refinement of the 3Rs [64].

All procedures were conducted in accordance with the Guide for the Care and Use of Laboratory Animals [65]. The initial experimental protocol was approved by the Scientific Ethics Committee of the University of La Frontera (File Number 067_24; 28 May 2024). Subsequent approval for the specific analysis of adipose tissue was granted by the same committee (File Number 116_24; 3 September 2024).

To generate the experimental litters, sexually mature virgin C57BL/6 females were paired with C57BL/6 males. Successful mating was confirmed by the presence of vaginal plugs, after which pregnant females were individually housed. During gestation and lactation, dams were provided the AIN-93G control diet, formulated by the American Institute of Nutrition to meet the nutritional requirements of this stage (Table 3) [66]. Diets were prepared by PRAGSOLUÇÕES Biociências (www.pragsolucoes.com.br (accessed on 1 June 2026)) and stored at −20 °C for the duration of the study to ensure optimal preservation.

Litters were monitored twice daily in the days preceding birth. The day of birth was designated as postnatal day 0 (PND 0). Sex identification was performed on PND 2 by visual identification of a darkly pigmented spot in the anogenital region, a feature specific to male neonates with dark pigmentation [67]. To standardize lactation conditions, litter size was adjusted to seven pups per dam.

### 4.2. Maternal Separation

Litters were randomly assigned to two experimental conditions. In the UM group, pups remained with their dams until conventional weaning at PND 21. In contrast, pups assigned to the MS group underwent prolonged daily separation during lactation and were weaned early. The MS protocol followed George et al. [66], in which pups were separated from the dam for 240 min daily from PND 2 to PND 5 and for 480 min daily from PND 6 to PND 16. Maternal separation was conducted as a single uninterrupted daily session during a fixed time window (09:00–13:00 from PND 2–5 and 09:00–17:00 from PND 6–16), rather than multiple sessions distributed throughout the day. Early weaning at PND17 constituted an integral component of the maternal separation model described by George et al. [68], whereas animals in the UM group underwent conventional weaning at PND21. During separation, pups were maintained at 32–34 °C on heating pads, while dams were housed in an adjacent room with ad libitum access to food and water. Animal hydration status and welfare were monitored daily following the protocol of Morton & Griffiths [69].

### 4.3. Post-Weaning Diet

**Figure 8:**
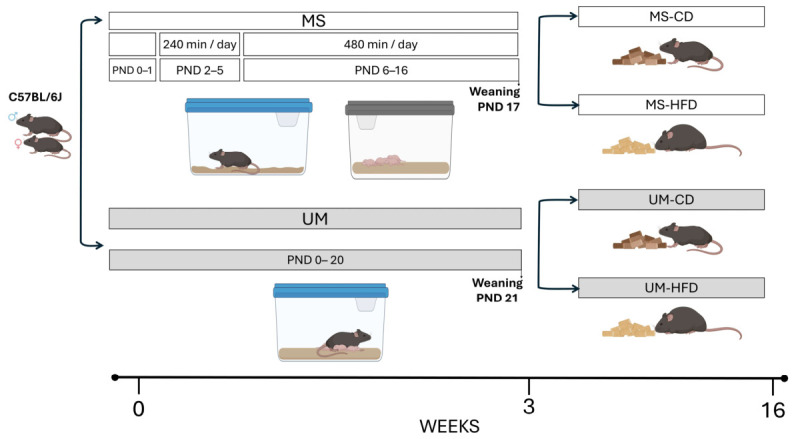
Experimental design of maternal separation and post-weaning dietary intervention. Schematic representation of the experimental timeline and group allocation. PND: postnatal day; UM: unmanipulated; MS: maternal separation; CD: control diet; HFD: high-fat diet; UM-CD: unmanipulated, control diet; MS-CD: maternal separation, control diet; UM-HFD: unmanipulated, high-fat diet; MS-HFD: maternal separation, high-fat diet. Selected graphical elements were incorporated from BioRender.com.

At weaning (PND 21 for UM and PND 17 for MS), one male per litter was randomly selected to constitute the experimental groups. Animals were subsequently assigned to one of four groups according to early-life condition and post-weaning diet: UM-CD, MS-CD, UM-HFD, and MS-HFD (*n* = 5 per group) (Figure 8).

To avoid variability related to the estrous cycle, only male animals were included, given that hormonal fluctuations can influence both behavior and food intake [70]. Each group received its designated diet for 16 weeks. The detailed composition of the diets is provided in Table 3 [71].

In accordance with the 3Rs principles of reduction and refinement [64], the number of animals per group was limited to five, which is considered sufficient for exploratory morphological studies. As noted by Cruz-Orive & Weibel [72], achieving consistent findings from a cohort of this size corresponds to an estimated probability of random occurrence equal to (1/2)^5^ = 0.03125, supporting the methodological adequacy of this sample size for exploratory studies. Furthermore, each animal was considered an independent biological replicate, while two histological sections per animal and ten microscopic fields per slide were used to obtain 100 representative measurements for each group, without artificially inflating the sample size. Additionally, the use of C57BL/6 mice maintained under standardized housing conditions, tissue processing, and morphological analysis, together with the evaluation of low-variability histopathological parameters, supports the suitability of this exploratory design.

### 4.4. Euthanasia

After 16 weeks of dietary intervention and a six-hour fasting period, euthanasia was performed by administration of a ketamine/xylazine overdose (240/30 mg/kg) [73]. Upon confirmation of loss of sensitivity, a midline abdominal incision was made to access the peritoneal cavity. The PGAT, RPAT, and MSAT, which represent anatomically and functionally distinct compartments of visceral adipose tissue, were then dissected.

### 4.5. Histological Processing and Staining

Following fixation (1.27 mol/L formaldehyde in 0.1 M phosphate buffer, pH 7.2) for 48 h at room temperature, samples were dehydrated and embedded in Paraplast Plus (Sigma-Aldrich Co., St. Louis, MO, USA). Serial sections, 4 µm thick, were obtained from the tissue blocks using a Leica^®^ RM 2255 microtome (Leica Biosystems, Deer Park, IL, USA). For histological analysis, ten sections per sample, spaced 150 µm apart, were collected. Sections were stained with hematoxylin and eosin (H&E) for evaluation of tissue architecture and with Picrosirius Red for identification of type I and III collagen fibers.

Histological images were acquired using a Leica^®^ DM750 microscope (Leica Microsystems, Heerbrugg, Switzerland) equipped with a Leica^®^ ICC50 HD digital camera (Leica Microsystems, Heerbrugg, Switzerland) and visualized on a ViewSonic^®^ LCD monitor (ViewSonic Corporation, Brea, CA, USA). To ensure comparability, all optical parameters, including illumination intensity, polarization angle, exposure time, and magnification, were held constant throughout image acquisition.

### 4.6. Histological Analysis of Inflammation in Adipose Tissue

Assessment of inflammatory alterations in adipose depots was performed on H&E-stained sections. Histological evaluation included the identification of mononuclear inflammatory infiltrates, adipocyte deformation, vascular alterations, and the presence of crown-like structures (CLS). For each animal, representative microscopic fields were obtained by systematic random sampling from selected sections and examined descriptively. Observations were recorded for each adipose depot to characterize histological patterns associated with maternal separation and dietary intervention. Observations were made using a Leica^®^ DM750 microscope (Leica Microsystems, Heerbrugg, Switzerland) equipped with a Leica^®^ ICC50 HD digital camera (Leica Microsystems, Heerbrugg, Switzerland) and were recorded descriptively for each adipose depot.

### 4.7. Quantification of Collagen Fibers

Type I and III collagen fibers in adipose depots were visualized by staining with 0.1% *w/v* Sirius Red F3BA (Sigma-Aldrich Co., St. Louis, MO, USA). Sections were incubated with Sirius Red for 1 h in saturated aqueous picric acid (Merck, Darmstadt, Germany), rinsed with 0.01 N hydrochloric acid (Merck, Darmstadt, Germany) for 2 min, washed with distilled water, and counterstained with Harris hematoxylin (Merck, Darmstadt, Germany) for 2 min. Finally, sections were dehydrated through ascending grades of alcohol, cleared in xylene (Merck, Darmstadt, Germany), and mounted with Entellan^®^ (Merck, Darmstadt, Germany).

For quantification, two histological sections per animal were analyzed (*n* = 5 animals per group). For each animal, eight images were acquired at 10× magnification by systematic random sampling within the adipose tissue parenchyma. Images were captured using a Leica^®^ DM750 microscope (Leica Microsystems, Heerbrugg, Switzerland) equipped with a Leica^®^ ICC50 HD digital camera (Leica Microsystems, Heerbrugg, Switzerland) and a polarized light system. Under these conditions, type I collagen fibers exhibited red-orange birefringence, while type III fibers displayed green-yellow birefringence. The area occupied by each fiber type was quantified by color segmentation using Image-Pro Premier 9.3 (Media Cybernetics, Warrendale, PA, USA), and the IOD of the signal was also recorded. Fibrosis was expressed as the Proportional Collagen Area (PCA), defined as the ratio of the area occupied by Picrosirius Red-stained collagen to the total tissue area analyzed. The areas corresponding to red-orange fibers (type I collagen) and green-yellow fibers (type III collagen) were also quantified separately.

### 4.8. Immunohistochemistry

Sections were rehydrated through a graded series of alcohols (Merck, Darmstadt, Germany) in decreasing concentrations, according to standard histological protocols, and immersed in distilled water for five minutes to restore hydration. Each section was then washed twice in 1 × PBS containing 0.2% Tween-20 (Sigma-Aldrich Co., St. Louis, MO, USA).

Endogenous peroxidase activity was blocked by incubating sections in H_2_O_2_ (*v*/*v*) (ab64264, Abcam, Cambridge, UK) for 20 min, followed by antigen retrieval using HistoReveal (ab103720, Abcam, Cambridge, UK) for 20 min at room temperature. After three buffer washes, nonspecific background was blocked with Protein Block (ab64264, Abcam, Cambridge, UK) for 30 min, followed by a second blocking step with Goat F(ab) Anti-Mouse IgG H&L (ab6668, Abcam, Cambridge, UK) for 60 min.

Sections were then incubated overnight at 4 °C in a humidified chamber with either Anti-leptin (ab16227, Abcam, Cambridge, UK) diluted 1:200 or Anti-UCP-1 (ab209483, Abcam, Cambridge, UK) diluted 1:500, as appropriate. Antibody dilutions were prepared in 3% BSA (Sigma-Aldrich Co., St. Louis, MO, USA) in 1 × PBS. Four buffer washes followed. Subsequently, sections were incubated with a biotinylated goat polyvalent antibody (ab64264, Abcam, Cambridge, UK) for 10 min at room temperature in a humidified chamber, followed by four buffer washes. Sections were then incubated with streptavidin peroxidase (ab64264, Abcam, Cambridge, UK) for 10 min at room temperature, followed by four buffer washes. Color development was performed by incubating sections with diaminobenzidine peroxidase (ab64264, Abcam, Cambridge, UK) for 2 min, followed by four buffer washes. Slides were counterstained with Harris hematoxylin (Sigma-Aldrich Co., St. Louis, MO, USA). Finally, sections were dehydrated through a graded series of alcohols (Merck, Darmstadt, Germany), cleared in xylene (Merck, Darmstadt, Germany), and mounted with Entellan^®^ (Merck, Darmstadt, Germany). Negative controls were processed by omitting the primary antibody and counterstained as described above. Images were acquired using a Leica^®^ DM750 microscope (Leica Microsystems, Heerbrugg, Switzerland) fitted with a Leica^®^ ICC50 HD digital camera (Leica Microsystems, Heerbrugg, Switzerland) using a 40× objective.

Two histological sections per adipose depot were analyzed for each animal (*n* = 5 per group). For each section, non-overlapping fields were acquired by systematic random sampling within the adipose parenchyma (12 fields per animal for leptin and 15 for UCP-1). Fields containing thick connective tissue septa, large-caliber vessels, sectioning artifacts, or section edges were excluded. DAB immunoreactivity was quantified by color segmentation using Image-Pro Premier 9.3 (Media Cybernetics, Warrendale, PA, USA), yielding the IOD. All images were acquired under constant illumination and exposure settings. Values per field were averaged to yield a single value per animal, which served as the experimental unit for statistical analysis.

### 4.9. Terminal Deoxynucleotidyl Transferase-Mediated Deoxyuridine Triphosphate (dUTP) Nick-End Labeling (TUNEL) Assay (Exploratory Evaluation)

DNA fragmentation was evaluated using the TUNEL-HRP-DAB kit (ab206386, Abcam, Cambridge, UK) on representative PGAT and MSAT samples. Due to limited reagent availability, the assay was performed on a small number of samples, including one representative animal per experimental group. Therefore, the observations derived from this assay are considered exploratory, and no statistical analysis was performed.

Sections were deparaffinized in xylene (Merck, Darmstadt, Germany) and rehydrated through a graded series of alcohols (Merck, Darmstadt, Germany), then washed in 1 × TBS (Merck, Darmstadt, Germany) for 5 min.

For permeabilization, sections were circumscribed with a hydrophobic marker and incubated with Proteinase K (Kit vial #1; 1 µL Proteinase K + 99 µL deionized water per sample) for 20 min at room temperature (RT), followed by a wash in 1 × TBS. Two additional washes in 1 × TBS were then performed. Endogenous peroxidase activity was quenched by incubating sections in 3% H_2_O_2_ in methanol (10 µL 30% H_2_O_2_ + 90 µL methanol; Merck, Darmstadt, Germany) for 20 min at RT, followed by one wash in 1 × TBS for 5 min.

For equilibration, TdT Equilibration Buffer (Kit vial #2) was applied for 10 min at RT, and excess liquid was removed. The labeling reaction mix was prepared immediately before use by combining 1 µL TdT Enzyme with 39 µL TdT Labeling Reaction Mix (Kit vial #4) per sample. Sections were incubated with this mixture under coverslips for 1 h 30 min at 37 °C in a humidified chamber. The reaction was stopped with Stop Buffer (Kit vial #5) for 5 min at 37 °C, followed by a wash in 1 × TBS.

For detection, sections were incubated with Blocking Buffer for 10 min at RT, then with Streptavidin-HRP (25×) diluted in Blocking Buffer (4 µL conjugate + 96 µL buffer per sample) for 30 min at RT in a humidified chamber, followed by a wash in 1 × TBS for 5 min.

Chromogenic development was performed using DAB solution (DAB Solution 1 + DAB Solution 2; 1:30 dilution) for 15–20 min, with color development monitored under the microscope, followed by two washes in deionized water.

Sections were counterstained with Methyl Green (Kit vial #10) for 1–3 min, dehydrated in 100% ethanol (2–4 changes), cleared in xylene (2–4 changes), and mounted with Entellan^®^ (Merck, Darmstadt, Germany).

For negative controls, TdT Enzyme was omitted from the reaction mixture. For positive controls, sections were treated with DNase I (Vivantis Technologies, Shah Alam, Selangor, Malaysia) for 20 min prior to labeling.

For the descriptive quantitative evaluation, the number of TUNEL-positive nuclei and the total number of adipocytes in each section were counted using Image-Pro Premier 9.3 (Media Cybernetics, Warrendale, PA, USA). The apoptotic index was expressed as the number of TUNEL-positive nuclei per 100 adipocytes.

### 4.10. Statistical Analysis

Data normality was assessed using the Shapiro–Wilk test, and homogeneity of variances was evaluated using the Brown-Forsythe test. Since the data met the assumptions of normality and homoscedasticity, two-way ANOVA was performed to assess the effects of MS, post-weaning diet, and their interaction. When significant differences were identified, Tukey’s post hoc multiple comparison test was applied. The animal was considered the biological replicate and the experimental unit for all analyses. Measurements were obtained from multiple technical observations within each animal (histological sections and systematically sampled microscopic fields), and values were averaged to generate a single representative value per animal prior to statistical analysis. Effect sizes (η^2^) were calculated for two-way ANOVA analyses and interpreted according to the proportion of total variance explained by each factor and interaction. All analyses were conducted using GraphPad Prism 10 (GraphPad Software, San Diego, CA, USA). Statistical significance was set at *p* < 0.05.

## 5. Conclusions

The findings of this study demonstrate that HFD-induced expansion of visceral adipose tissue reconfigures stromal architecture and adipocyte signaling in a depot-specific manner, and that this response is modulated by early-life metabolic programming. PGAT exhibited a predominantly adaptive pattern, RPAT was sensitive to the convergence of early-life programming and nutritional challenge, and MSAT emerged as the most vulnerable depot, displaying the most pronounced stromal remodeling and leptin dysregulation. Residual UCP-1 immunoreactivity in MS animals may reflect altered adipose tissue metabolic responsiveness associated with early-life stress exposure. Collectively, these results provide histological and immunohistochemical evidence that maternal separation is associated with depot-specific alterations in the response of visceral adipose tissue to an obesogenic nutritional challenge.

## Figures and Tables

**Table 1:** Two-way ANOVA evaluating the effects of diet, maternal separation, and their interaction on collagen morphometric parameters in visceral adipose depots.

Adipose Depot	Collagen Morphometric Parameter	Factors		
		Diet	Maternal Separation	Diet × MS Interaction
PGAT	Total Collagen Area (%)	F(1,16) = 0.0001; *p* = 0.9921	F(1,16) = 0.207; *p* = 0.6557	F(1,16) = 1.201; *p* = 0.2894
	Normalized Proportion of Collagen type I Fibers	F(1,16) = 0.470; *p* = 0.5025	F(1,16) = 2.948; *p* = 0.1053	F(1,16) = 0.0014; *p* = 0.9712
	IOD of Collagen type I Fibers	**F(1,16) = 6.030; *p* = 0.0259**	F(1,16) = 1.783; *p* = 0.2005	F(1,16) = 1.003; *p* = 0.3315
	IOD of Collagen type III Fibers	**F(1,16) = 8.284; *p* = 0.0109**	F(1,16) = 0.142; *p* = 0.7113	F(1,16) = 0.055; *p* = 0.8175
RPAT	Total Collagen Area (%)	**F(1,16) = 5.665; *p* = 0.0301**	F(1,16) = 1.592: *p* = 0.2252	**F(1,16) = 11.17; *p* = 0.0041**
	Normalized Proportion of Collagen type I Fibers	F(1,16) = 1.606; *p* = 0.2232	**F(1,16) = 16.15; *p* = 0.0010**	F(1,16) = 2.358; *p* = 0.1442
	IOD of Collagen type I Fibers	**F(1,16) = 6.481; *p* = 0.0216**	**F(1,16) = 6.566; *p* = 0.0209**	**F(1,16) = 12.03; *p* = 0.0032**
	IOD of Collagen type III Fibers	**F(1,16) = 46.07; *p* < 0.0001**	**F(1,16) = 80.54; *p* < 0.0001**	**F(1,16) = 52.23; *p* < 0.0001**
MSAT	Total Collagen Area (%)	F(1,16) = 1.404; *p* = 0.2533	**F(1,16) = 35.11; *p* < 0.0001**	F(1,16) = 0.958; *p* = 0.3422
	Normalized Proportion of Collagen type I Fibers	**F(1,16) = 5.902; *p* = 0.0273**	F(1,16) = 0.0003; *p* = 0.9853	F(1,16) = 1.716; *p* = 0.2088
	IOD of Collagen type I Fibers	F(1,16) = 3.209; *p* = 0.0922	**F(1,16) = 16.81; *p* = 0.0008**	F(1,16) = 0.338; *p* = 0.5694
	IOD of Collagen type III Fibers	F(1,16) = 4.261; *p* = 0.0556	**F(1,16) = 20.06; *p* = 0.0004**	F(1,16) = 0.037; *p* = 0.8495

PGAT: perigonadal adipose tissue; RPAT: retroperitoneal adipose tissue; MSAT: mesenteric adipose tissue. IOD: Integrated Optical Density. *p* < 0.05 is considered statistically significant. **Significant effects are in bold letters.**

**Table 2:** Two-way ANOVA evaluating the effects of diet, maternal separation, and their interaction on Leptin Immunoreactivity in visceral adipose depots from male C57BL/6 mice.

Adipose Depot	Leptin Immunoreactivity (IOD)		
	Diet	Maternal Separation	Diet × MS Interaction
PGAT	F(1,16) = 0.096; *p* = 0.7611	F(1,16) = 0.299; *p* = 0.5922	F(1,16) = 0.755; *p* = 0.3978
RPAT	F(1,16) = 0.195; *p* = 0.6646	F(1,16) = 2.675: *p* = 0.1215	**F(1,16) = 4.753; *p* = 0.0482**
MSAT	F(1,16) = 3.152; *p* = 0.0949	F(1,16) = 0.196; *p* = 0.6638	**F(1,16) = 37.01; *p* < 0.0001**

PGAT: perigonadal adipose tissue, RPAT: retroperitoneal adipose tissue, MSAT: mesenteric adipose tissue, IOD: Integrated Optical Density. *p* < 0.05 is considered statistically significant. **Significant effects are in bold letters**.

**Table 3:** Diets were administered according to the experimental group. Control diet (CD) and high-fat diet (HFD). Vitamin and mineral mixes were formulated according to the AIN-93G standard of the American Institute of Nutrition for rodents [66]. The high-fat diet provided 49% of the energy through lipids.

Ingredients	CD	HFD
Casein (>85% protein)	200.0	230.0
L-cystine (g/kg)	3.0	3.0
Cornstarch (g/kg)	529.486	299.472
Sucrose (g/kg)	100.0	100.0
Soybean oil (g/kg)	70.0	70.0
Lard (g/kg)	-	200.0
Fiber (g/kg)	50.0	50.0
Vitamin mixture (g/kg)	10.0	10.0
Mineral mixture (g/kg)	35.0	35.0
Choline bitartrate (g/kg)	2.5	2.5
Antioxidant (g/kg)	0.014	0.028
Total (g)	1000.0	1000.0
Energy (kcal/g)	3.95	4.95
Carbohydrate (% Energy)	64.0	32.0
Protein (% Energy)	19.0	19.0
Lipid (% Energy)	17.0	49.0

## Data Availability

The raw data supporting the conclusions of this article will be made available by the authors on request.

## Abbreviations

The following abbreviations are used in this manuscript:

CD	Control diet
CLS	Crown-like structure
ECM	Extracellular matrix
HFD	High-fat diet
IOD	Integrated optical density
MS	Maternal separation
MSAT	Mesenteric adipose tissue
MS-CD	Maternal separation group with control diet
MS-HFD	Maternal separation group with a high-fat diet
PGAT	Perigonadal adipose tissue
RPAT	Retroperitoneal adipose tissue
UCP-1	Uncoupling protein 1
UM	Unmanipulated
UM-CD	Unmanipulated group with control diet
UM-HFD	Unmanipulated group with a high-fat diet
VAT	Visceral adipose tissue
